# A Novel Space–Time Coding Echo Separation Scheme with Orthogonal Frequency Division Multiplexing Chirp Waveforms for Multi-Input Multi-Output Synthetic Aperture Radar

**DOI:** 10.3390/s25061717

**Published:** 2025-03-10

**Authors:** Kai Yao, Chang Liu

**Affiliations:** 1 Aerospace Information Research Institute, Chinese Academy of Sciences, Beijing 100094, China; 2 School of Electronic, Electrical and Communication Engineering, University of Chinese Academy of Sciences, Beijing 100049, China

**Keywords:** space–time coding, MIMO-SAR, OFDM waveform, SNR

## Abstract

Multi-input Multi-output Synthetic Aperture Radar (MIMO-SAR) systems significantly improve the performance of traditional SAR systems by providing more system freedom. However, in the working mode of the simultaneous transceiver, each receiving antenna will receive the scattered echoes of all transmitting antennas, resulting in the overlapping of echo data and serious related interference, which becomes the main obstacle to the further development and application of MIMO-SAR system. Therefore, achieving effective echo separation is the key technical challenge faced by the MIMO-SAR system. Space–time coding (STC) uses multiple dimensions, such as space, time, and frequency. Through the process of encoding and decoding in these dimensions, channel information can be obtained, and echo separation can be realized. STC is suitable for MIMO-SAR system on different platforms, such as airborne, and has wide applicability. When the traditional scheme uses STC as a coding scheme, it is generally limited by the two-dimensional sending and receiving matrix of Alamouti code. To solve this problem, a new STC scheme based on complex orthogonal matrix design is proposed in this paper. The scheme can form a multidimensional orthogonal STC matrix, recover the superposed signal by echo decoding, and improve the echo signal-to-noise ratio (SNR) of MIMO-SAR. In addition, the use of orthogonal frequency division multiplexing (OFDM) waveform can further reduce cross-correlation interference to achieve effective separation of MIMO-SAR echoes. The effectiveness of the waveform scheme is verified by numerical experiments.

## 1. Introduction

With the rapid development of radar technology, driven by advances in areas such as precise imaging [1], target detection and recognition [2,3], target reconstruction [4], and data fusion, the application of radar systems has gradually expanded, becoming an important technology in modern scientific research and engineering and promoting progress in the field of remote sensing. As the demand for high-precision imaging and target detection continues to grow, radar waveform design can effectively overcome the technical challenges that conventional signal processing methods struggle to solve [5], thus further promoting the realization and application of new SAR systems. MIMO-SAR, as one of the most developed parts of the SAR system, through multiple transceiver units working at the same time, achieves a similar radar collaboration effect, expanding the performance of the SAR system and the degree of freedom [6]. MIMO-SAR generally adopts the operation mode of simultaneous transmission and reception. However, in this mode, each receiving antenna will receive the scattered echoes of all transmitting antennas, resulting in the mutual aliasing of echo data and serious cross-correlation interference [7]. Therefore, echo separation is one of the main technical challenges faced by MIMO-SAR systems.

The development of MIMO-SAR technology has always focused on how to apply MIMO technology to solve SAR problems [8,9]. Traditional single-dimensional diversity methods, such as time division and frequency division, cannot maximize the utilization of MIMO-SAR system resources. With the deepening of research, the MIMO-SAR echo separation scheme based on simultaneous and co-frequency waveforms has gradually become the main research direction. Since the information transmission process of radar is similar to the receiving process and the structure of the communication system, for the SAR system, only the transmitted signal and the received signal are known, and what needs to be obtained is the channel response, which is the scattering characteristics and position information of the target so that STC technology can be applied to the MIMO-SAR waveform after certain adjustment and improvement. STC has been widely used and developed in MIMO radar [10,11,12,13], including realizing new radar functions, such as radar communication integration, etc. By encoding and decoding in spatial and time–frequency domains, echo separation of waveform signals can be realized without increasing the complexity of system hardware, which is suitable for MIMO-SAR systems on different platforms, such as airborne [14].

It is proposed that the Alamouti coding form can be used to achieve the encoding and decoding operation of a two-dimensional transceiver unit, so as to support echo separation, which establishes the technical route of using the STC method to realize the MIMO-SAR waveform [15]. On this basis, a series of waveform design schemes centering on Alamouti code are proposed successively. Due to the presence of autocorrelation and cross-correlation interference between certain waveforms in the radar echo after STC [16], in [17], the authors effectively reduced waveform interference by utilizing multi-phase codes (MPC) with orthogonality and extending the Alamouti code. However, the exploitation of waveform degrees of freedom remained limited. To address the issue of increased interference after echo decoding due to significant changes in the radar transmission channel, the approach in [18] simplified the Alamouti code to eliminate its effects on time-varying channels. Nevertheless, this encoding scheme suffers from poor scalability and struggles to improve the SNR. In [19], two Alamouti cycles were implemented within one transmission duration, and DBF technology was used for echo separation to minimize time-varying effects. However, this also introduced new interference and increased hardware complexity. The authors of [20,21] applied the Alamouti code to different Doppler center frequencies and employed Doppler filters for signal separation, increasing the dimensionality of the code. However, this method requires a high repetition frequency for the transmitted signal. In [22], to further enhance the orthogonality of STC, a multi-frame STC method based on the Alamouti code was proposed. However, this approach also consumes more transmission pulses and decoding cycles, leading to a reduction in computational efficiency and potential interference risks. Although Alamouti code can effectively achieve echo separation, its two-dimensional structure limits the scale of the separated signal, which limits the further application of STC.

The combination of STC with other coding or waveforms can effectively solve diverse functional requirements; the authors of [23] combined STC and phase-coding, improving the freedom of the waveform design scheme. OFDM waveforms gradually show advantages in MIMO radar because of their orthogonality and design flexibility [24,25,26,27]. The use of the OFDM waveform can effectively overcome the potential signal interference of a MIMO-SAR system and become an effective supplement to the STC scheme [28,29,30,31,32,33]. Therefore, based on the analysis of the above-mentioned research, this study proposes a new STC scheme with OFDM waveform from the perspective of complex orthogonal matrix design to separate different MIMO-SAR echoes. This approach overcomes the dimensional limitations of the traditional Alamouti scheme. By establishing a complex orthogonal matrix, an arbitrary dimensional transceiver codec scheme is formed. In order to meet the requirements of the number of waveforms in the multi-dimensional coding scheme and overcome the relevant interference caused by errors in the decoding process, OFDM waveforms are introduced, and the signal correlation performance is optimized to further improve the orthogonality of the transmitted waveforms. Through the design framework of STC and transmitting waveform, the overall effect of MIMO-SAR echo separation can be further improved, thus improving the performance of the radar system.

## 2. Signal Model

### 2.1. Preliminaries

**Figure 1:**
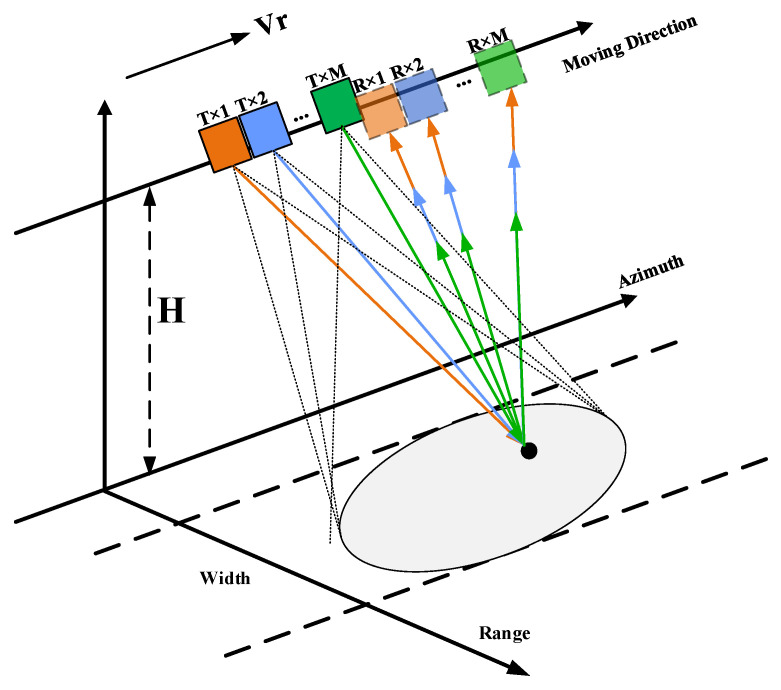
Co-located MIMO-SAR operating system.

In MIMO-SAR systems, the received signals are a mixture of echoes coming from multiple transmit antennas. The geometric structure of the MIMO-SAR system is shown in Figure 1. The uniform linear array antenna is distributed with *M* transceiver units along the azimuth, and each unit simultaneously receives echo signals from different transmitting units with waveforms. Suppose that the echo received by the *n*-th antenna from the m antenna can be represented as *r_mn_*. Then, the received signal received by the *n*-th antenna can be expressed as the sum of signals transmitted from all *M* antennas:

Let the mixed echo received by the *n*-th antenna be represented as

(1) $$r_{n}(t,\eta )=\sum_{m=1}^{M}r_{mn}(t,\eta )=\sum_{m=1}^{M}h_{mn}s_{m}+n_{n}$$

where *r_n_* is the received signal vector at the *n*-th antenna, *h_mn_* is the channel gain, representing the propagation characteristics from the *m*-th transmiting antenna to the *n*-th receiving antenna. *s_m_* is the transmitted signal from the *m*-th antenna. *n* is the noise vector at the *n*-th receiver antenna, typically modeled as Gaussian noise.

The goal of echo separation is to individually obtain the echoes from different transmitting antennas:

(2) $$\;r_{n,1}(t,\eta ),\ldots ,r_{n,M}(t,\eta )$$

Echo separation can be achieved through various methods, each with different requirements for the transmitted signals. Both time-division and frequency-division approaches can be used to separate waveforms at different transmit and receive times or in different frequency bands. However, these methods inevitably sacrifice the system resources of MIMO-SAR. To maximize the performance of MIMO-SAR, the ultimate trend is to use simultaneous and co-frequency signals for transmission. The key to separating simultaneous and co-frequency signals lies in achieving orthogonality of the waveforms. However, strictly orthogonality is difficult to achieve in practical systems. Therefore, to improve the orthogonality of the transmitted and received waveforms, multi-domain design and processing is an effective solution.

Conventional one-dimensional orthogonal signals are limited in degrees of freedom, meaning the number of variables in the orthogonal criterion is limited. However, by simultaneously utilizing time, space, frequency, and coding dimensions, and introducing multiple waveform variables into the orthogonality criteria, it is possible to disperse multiple signals across a multi-dimensional space. This can be achieved by introducing additional degrees of freedom, leading to the construction of a multi-dimensional waveform orthogonality criterion, which satisfies the requirement for separating aliased signals. The orthogonality of the encoded signals ensures that, despite the interference caused by multiple transmit and receive antennas, the signals can still be separated and decoded correctly. For two encoded signals *x*_1_(*t*,*s*,*c*) and *x*_2_(*t*,*s*,*c*), their inner product is calculated over time as

(3) $$\chi (\tau ,0)=\int_{-\infty }^{\infty }x_{1}(t,s,c)\cdot x_{2}(t,s,c)dt=\delta (0<\delta \leq 1)$$

where *χ*(*τ*, 0) represents the inner product between the signals. *δ* is the Dirac delta function, which enforces the condition of orthogonality between the two signals. The presence of the function indicates that the two signals are orthogonal under the given conditions. This is the basic principle—that STC can be applied to MIMO-SAR waveform design. The key idea of STC is to map the transmitted signal into a multi-dimensional space, where signals are encoded over both time and space (and possibly frequency). This encoding scheme helps in maintaining orthogonality between different signals, ensuring that they can be successfully decoded at the receiver. In the context of STC-MIMO-SAR systems, the transmitted signal vector *s* can be expressed as

(4) s=Cd

*s* = [*s*_1_, *s*_2_, …, *s_M_*]^T^ is the vector of transmitted signals from the *M* antennas. *d* is the vector of symbols to be transmitted, each corresponding to a time slot or frequency bin. *C* = [*c*_1_, *c*_2_, …, *c_n_*] is the STC matrix of size *M* × *T*, where *M* is the number of transmit antennas, and *T* is the number of time slots or channels used for transmission. The matrix *C* is designed to ensure that signals from different antennas remain orthogonal in the multi-dimensional space:

(5) $$C^{H}C=I$$

By constructing multidimensional coding waveform in the airspace and comprehensively utilizing the dimensions of time, space, and coding, STC waveform is formed, and different waveforms are transmitted in the connected time periods. By using the echoes received at slow time, the parallel observation channel data are decomposed to meet the orthogonality requirement of echo separation. The key problem in the application of STC technology in MIMO-SAR is that for MIMO communication, the information from the source is expected to be obtained, which allows the coding scheme to have a certain bit error rate. For MIMO-SAR, because it is the electromagnetic wave detection process of radar, the codecs process is to obtain the scattering information of ground objects, which is the channel information. Therefore, SAR codec requires the signal to achieve full orthogonality, which is the main reason that traditional SAR uses Alamouti encoding scheme.

### 2.2. Alamouti Coding Scheme

**Figure 2:**
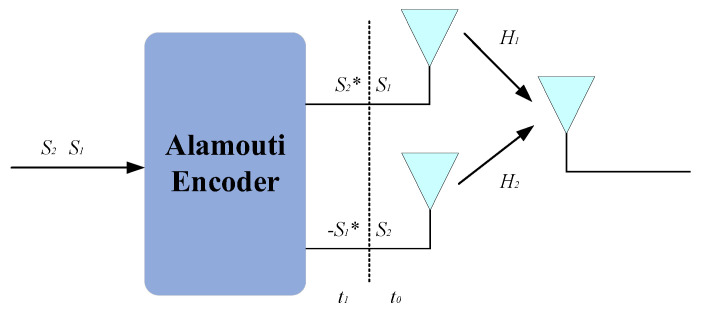
Alamouti−Coded Reception Model.

Classical STC-MIMO-SAR schemes originate from one of the key technologies in third-generation wireless communications, the Alamouti coding scheme. In SAR, the entire transmitted waveform is treated as the object of encoding and decoding. Utilizing a 2×2 MIMO antenna configuration, the Alamouti coding scheme transmits waveforms that are conjugate and complex–conjugate pairs over two pulses. Through the encoding and decoding processes of the transmitted and received waveforms, the target transfer function embedded in the channel is obtained, thereby achieving echo separation. The geometric model of a 2-transmit, 2-receive SAR system based on the Alamouti-coded STC scheme is shown in Figure 2.

In the first period, the two transmitting antennas, T_x1_ and T_x2_, emit signals *s*_1_(*t*) and *s*_2_(*t*) with a pulse width of *τ*, respectively. The receiving antennas, Rx1 and Rx2, simultaneously capture the echoes. In the next period, T_x1_ and T_x2_ emit signal s*_2_(−*t*) and −s*_1_(−*t*) with the same pulse width, respectively. This process is then repeated continuously. This matrix representation facilitates the encoding and decoding processes, ensuring the orthogonality required for effective echo separation in the MIMO-SAR system.

(6) $$S=\left[\begin{array}{cc} S_{1} & S_{2}\\ S_{2}^{*} & -S_{1}^{*} \end{array}\right]$$

In (6), each element of the matrix represents a transmitted signal. Specifically, each row represents a transmission queue, where *S*_1_ and *S*_2_* form the first transmission queue, and *S*_2_ and −*S*_1_ form the second transmission queue. The two transmit elements operate in the first and second Pulse Repetition Intervals (PRIs).

Let *H*_1_ and *H*_2_ denote the channel responses that need to be estimated. In communication applications, the transmitted signal *S* is detected based on the estimated channel response *H* and the received signal *R*.

(7) $$H=\left[\begin{array}{c} H_{1},H_{2} \end{array}\right]$$

However, in SAR applications, the transmitted and received signals are known, and the objective is to detect the channel response *H*, which encompasses the scattering characteristics and position information of the target. Consequently, the decoding matrix *D* can be constructed to facilitate this detection process.

(8) $$D=S^{H}=\left[\begin{array}{cc} S_{1}^{*} & S_{2}\\ S_{2}^{*} & -S_{1} \end{array}\right]$$

In Equation (8), the inverted channel characteristics can be derived as follows:

(9) $$\left[\begin{array}{l} R_{1}^{\prime }\\ R_{2}^{\prime } \end{array}\right]=D\cdot \left[\begin{array}{l} R_{1}\\ R_{2} \end{array}\right]=\left[\begin{array}{cc} S_{1}^{*} & S_{2}\\ S_{2}^{*} & -S_{1} \end{array}\right]\cdot \left[\begin{array}{cc} S_{1} & S_{2}\\ S_{2}^{*} & -S_{1}^{*} \end{array}\right]\cdot \left[\begin{array}{c} H_{1}\\ H_{2} \end{array}\right]=\left[\begin{array}{l} \left({\left|S_{1}\right|}^{2}+{\left|S_{2}\right|}^{2}\right)H_{1}\\ \left({\left|S_{1}\right|}^{2}+{\left|S_{2}\right|}^{2}\right)H_{2} \end{array}\right]$$

Since, for SAR systems, the transfer function of target information is contained within the channel, by inverting and calculating the channel, the output corresponding to the equivalent phase center can be obtained, and imaging processing can be performed on the obtained results. For this process, it utilizes the conventional structure of Alamouti, which imposes a limitation on the number of transmitted and received signals, restricting them to two dimensions.

## 3. STC Scheme

To overcome the dimensional limitations imposed by the Alamouti structure on the encoding matrix, a detailed analysis was conducted on its applicability to MIMO-SAR. It is crucial to emphasize that the driving factor in this process is not simply relying on the encoding arrangement that satisfies the Alamouti structure, but rather on the complex orthogonality inherent in space–time block codes. As discussed in previous sections, the key to applying Alamouti coding in MIMO-SAR lies in leveraging its complex orthogonal properties. Building upon this foundation, this paper proposes a complex orthogonal STC method, which enables the design of encoding schemes for transceiver matrices of arbitrary dimensions [34,35,36,37]. The codec process under this proposed scheme involves progressively designing a complex orthogonal matrix by adding rows and columns to the coding matrix, extending the matrix in a regular pattern to maintain its orthogonal properties. This approach demonstrates that the process can be extended from *n* to *n* + 1 dimensions, providing an effective method for constructing complex orthogonal matrices.

Let *X* be a *p* × *n* matrix, where its elements are linear combinations of the variables *x*_1_, *x*_2_, …, *x_n_* and their complex conjugates *x*_1_*, *x*_2_*, …, x_n_*. For STC of any dimension, the coding symbols can be represented as

(10) $$\left(0,x_{1},x_{2},\ldots ,x_{k},-x_{1},-x_{2},\ldots ,-x_{k},x_{1}^{*},x_{2}^{*},\ldots ,x_{n}^{*},-x_{1}^{*},-x_{2}^{*},\ldots ,-x_{n}^{*}\right)$$

These coding symbols must satisfy the orthogonality condition:

(11) $$X^{H}X=\left({\left|x_{1}\right|}^{2}+{\left|x_{2}\right|}^{2}+\cdots +{\left|x_{u}\right|}^{2}\right)I$$

where (.)*^H^* denotes the complex conjugate transpose, and *I* is an *n* times identity matrix. It is evident that when *n* = 2, the matrix reduces to the Alamouti code.

To establish a scalable complex orthogonal matrix, submatrices need to be constructed based on the elements of the matrix. Through the properties satisfied by these submatrices, the overall complex orthogonal matrix can be formed. For clarity, we define the following rules for constructing the submatrices. Let *X_n_* be a complex orthogonal matrix of dimension *n*, where the number of transmitting antennas is *n*. Let ${\bar{X}}_{n}$ be a column vector of length *p*, where the elements are the transmitted waveform symbols from *X_n_*, and $X_{n}^{\prime }$ be a column vector of length *q.* Additionally, let *Φ* be a complex orthogonal matrix that has the same structure as the *X_n_*, and its elements are derived from *X_n_*, but it has a smaller dimension and serves as a supplement to facilitate the construction of a new matrix. In essence, the underlying complex orthogonal matrix and its corresponding column vectors are defined These submatrices help establish a generalized design methodology for constructing complex orthogonal matrices.

Define the constructed complex orthogonal matrix; let matrix *A* be $A_{1}=\left[\begin{array}{l} A_{11}\;A_{12}\\ A_{21}\;A_{22} \end{array}\right]$, *A*_11_ and *A*_22_, and *A*_21_ and *A*_12_ have the same set of nonzero complex variables, whose subscript represents the number of transmit antennas, and the elements in the matrix are composed of the above coding symbols. It should be satisfied that the subscript number only appears at most once in the same row or column. The form of the matrix is divided according to the different types of elements. The elements of the matrix consist of sequential signal symbols, complex conjugations, and empty elements. Then, the following $\bar{A}=\left[\begin{array}{c} {\left(-1\right)}^{k}A_{11}\;{\left(-1\right)}^{l}A_{12}\\ {\left(-1\right)}^{m}A_{21}\;{\left(-1\right)}^{n}A_{22} \end{array}\right]$ is also a complex orthogonal design if *k + l + m + n* is even [38,39,40].

In order to ensure that the obtained matrix satisfies the complex orthogonality condition, it is necessary to ensure that any column in the designed matrix is orthogonal to all other columns in it. Therefore, when extending the matrix, its corner matrix elements should be extended in a way that satisfies the complex orthogonality condition so that the extended columns are also complex orthogonality, respectively. Let *n =* 2*k* − 1, *k* = 1; 2; *…*; then, the starting matrix is

(12) $$X_{n+1}=\left[\begin{array}{cc} X_{n}(1) & {\bar{X}}_{n}(2)\\ X_{n}(2) & {\left(-1\right)}^{k}{\bar{X}}_{n}(1) \end{array}\right]$$

When *n = n +* 2, the corner element is extended to 3 in order, and the corner element still needs to meet the structure of *n*, so the position and symbol of the new element in each column can be determined, and the position of the other two elements can be determined according to the requirement that the same sequence number can only appear once in the column so as to determine the final complex orthogonal matrix structure.

(13) Xn+2=Xn(1) X¯n(2)Xn(2) (−1)kX¯n(1)X¯n(3)ϕ¯1,n(4)Xn(3) −ϕ¯1,n(4)ϕ1,n(4) Xn′(3)(−1)kX¯n(1)−Xn′(2)

When *n = n +* 3, the resulting matrix should be derived by extending the previous matrix in the same structural form, with an increase in the number of variables while maintaining the original structure to preserve the complex orthogonality. For the newly added columns, representing additional transmit units, their transmitted waveforms should maintain orthogonality with the corresponding elements of other columns, thereby forming a new, complex orthogonal matrix.

(14) $$X_{n+3}=\left[\begin{array}{cc} X_{n+2}(1) & {\bar{X}}_{n+2}(2)\\ X_{n+2}(2) & {\left(-1\right)}^{k+1}{\bar{X}}_{n+2}(1) \end{array}\right]=\left[\begin{array}{cc} \begin{array}{cc} \begin{array}{l} X_{n}(1)\;{\bar{X}}_{n}(2)\\ X_{n}(2)\;{\left(-1\right)}^{k}{\bar{X}}_{n}(1) \end{array} & \begin{array}{l} {\bar{X}}_{n}(3)\\ {\bar{\phi }}_{1,n}(4) \end{array}\\ \begin{array}{l} X_{n}(3)\;-{\bar{\phi }}_{1,n}(4)\\ \phi_{1,n}(4)\;X_{n}^{\prime }(3) \end{array} & \begin{array}{l} {\left(-1\right)}^{k}{\bar{X}}_{n}(1)\\ -X_{n}^{\prime }(2) \end{array} \end{array} & \begin{array}{l} {\left(-1\right)}^{k}{\bar{\phi }}_{1,n}(8)\\ {\bar{X}}_{n}(7)\\ -{\bar{X}}_{n}(6)\\ X_{n}^{\prime }(5) \end{array}\\ \begin{array}{cc} \begin{array}{l} X_{n}(5)\;{\bar{X}}_{n}(6)\\ X_{n}(6)\;{\left(-1\right)}^{k}{\bar{X}}_{n}(5) \end{array} & \begin{array}{l} {\bar{X}}_{n}(7)\\ {\bar{\phi }}_{1,n}(8) \end{array}\\ \begin{array}{l} X_{n}(7)\;-{\bar{\phi }}_{1,n}(8)\\ \phi_{1,n}(8)\;X_{n}^{\prime }(7) \end{array} & \begin{array}{l} {\left(-1\right)}^{k}{\bar{X}}_{n}(5)\\ -X_{n}^{\prime }(6) \end{array} \end{array} & \begin{array}{l} -{\bar{\phi }}_{1,n}(4)\\ {\left(-1\right)}^{k+1}{\bar{X}}_{n}(3)\\ {\left(-1\right)}^{k}{\bar{X}}_{n}(2)\\ {\left(-1\right)}^{k+1}X_{n}^{\prime }(1) \end{array} \end{array}\right]$$

Then, for *n +* 1, it is constructed in such a way that each of its parts is also complex orthogonal, and its rows and columns are orthogonal, so eventually, *N* + 2 is also orthogonal, and by induction, it follows that for *N* > 2, the method can obtain a complex orthogonal matrix. Through the above process, we formed the design result of a complex orthogonal matrix and completed the construction of STC scheme. Next, the process of encoding and decoding is explained.

**Figure 3:**
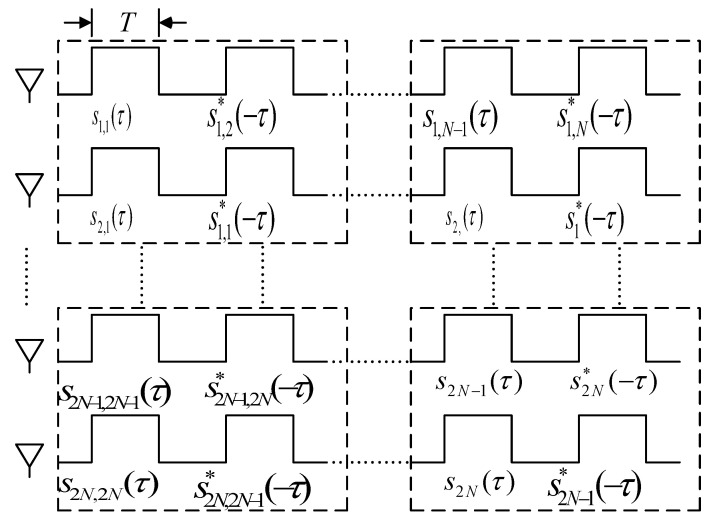
Timing distribution of arbitrary dimension STC.

According to the temporal distribution in Figure 3, for a MIMO-SAR system with *N* subarrays along the azimuth direction, during the slow time *η* = *kT*, which corresponds to the *k*-th PRI, the *n*-th transmit subarray Tx emits the signal *s_n,k_(τ)*, where *τ* denotes the fast time. Let *f_r_* be the range frequency, and *S_n,k_(f_r_)* be the frequency domain representation of the waveform transmitted by the *n*-th transmit element at time *k*. Let *H_n,m_(f_r_)* be the channel frequency response between the *n*-th transmit element and the *m*-th receive element. 

Additionally, let *n_m,k_(f_r_)* be the additive Gaussian noise of the radar system at time *k*. The total transmitted waveform for the *k*-th PRI can be expressed as

(15) $$s_{k}(f_{r})={\left[S_{1,k}(f_{r}),S_{2,k}(f_{r}),\ldots S_{N,k}(f_{r})\right]}^{T}$$

The echo received by the *m*-th subarray *R_x_* over *k* transmission cycles can be expressed as

(16) $$R_{m,k}\left(f_{r}\right)=h_{m}^{T}\left(f_{r}\right)s_{k}\left(f_{r}\right)+n_{m,k}\left(f_{r}\right)$$

The echo received by the *m*-th receive subarray *R_x_* over *k* transmission cycles can be expressed as

(17) $$\begin{array}{l} R_{m}^{T}\left(f_{r},f_{a}\right)=\left[H_{m,1}\left(f_{r},f_{a}\right)\ldots H_{m,2k}\left(f_{r},f_{a}\right)\right]\\ \times \left[\begin{array}{ccc} S_{1}(f_{r}), & S_{2}^{*}(f_{r})e^{j2\pi f_{a}T}, & \ldots \\ S_{2}(f_{r}), & -S_{1}^{*}(f_{r})e^{j2\pi f_{a}T}, & \ldots \\ \vdots & \vdots & \\ S_{2N-1}(f_{r}), & S_{2N-1}^{*}(f_{r})e^{j2\pi f_{a}T},\; & \ldots \\ S_{2N}(f_{r}), & -S_{2N}^{*}(f_{r})e^{j2\pi f_{a}T}, & \ldots \end{array}\right]+n_{m}^{T}\left(f_{r},f_{a}\right) \end{array}$$

During the reception phase, the ground echoes are received by *N* different receiver subarrays *R_x_*, forming multiple receive phase centers in each PRI. The echo matrix for all channels can be written as

(18) $$r_{m}^{itT}\left(f_{r}\right)=h_{m}^{T}\left(f_{r}\right)S\left(f_{r}\right)+n_{m}^{T}\left(f_{r}\right)$$

The decoding matrix *D(f_r_,f_a_)* is constructed as

(19) $$D_{m}(f_{r},f_{a})=\left[\begin{array}{lll} S_{1}^{*}(f_{r}), & \;S_{2}^{*}(f_{r}), & \ldots \\ S_{2}(f_{r})e^{-2\pi f_{a}T}, & -S_{1}(f_{r})e^{-2\pi f_{a}T}, & \ldots \\ \;\vdots & \;\;\vdots & \\ S_{2N-1}^{*}(f_{r}), & \;S_{2N}^{*}(f_{r}), & \ldots \\ S_{2N}(f_{r})e^{-2\pi f_{a}T}, & -S_{2N-1}(f_{r})e^{-2\pi f_{a}T}, & \ldots \end{array}\right]$$

By multiplying the received echo matrix with the decoding matrix, the decoded result can be obtained as

(20) $$\begin{array}{l} \begin{array}{c} r_{D,m}^{T}\left(f_{r}\right)=r_{m}^{T}\left(f_{r}\right)D\left(f_{r}\right)\\ =h_{m}^{T}\left(f_{r}\right)S\left(f_{r}\right)D\left(f_{r}\right)+n_{m}^{T}\left(f_{r}\right)D\left(f_{r}\right) \end{array}\\ =\left[H_{m,1}\left(f_{r},f_{a}\right)\ldots H_{m,n}\left(f_{r},f_{a}\right)\right]\\ \times \left[\begin{array}{ccc} \sum_{i=1}^{K}{\left|S_{i}\left(f_{r}\right)\right|}^{2} & \ldots & 0\\ \vdots & \ddots & \vdots \\ 0 & \ldots & \sum_{i=1}^{K}{\left|S_{i}\left(f_{r}\right)\right|}^{2} \end{array}\right]+n_{md}^{T}(f_{r},f_{a}) \end{array}$$

It can be seen that for single-echo transmission and reception, the echo signal is

(21) $$S_{n_{rc}}\left(t_{a},f_{r}\right)=\left[\begin{array}{c} H_{1,n}\left(t_{a},f_{r}\right)\cdot {\left|S_{k,1}\right|}^{2}\\ H_{2,n}\left(t_{a},f_{r}\right)\cdot {\left|S_{k,2}\right|}^{2}\\ \vdots \\ H_{M,n}\left(t_{a},f_{r}\right)\cdot {\left|S_{k,M}\right|}^{2} \end{array}\right]+\left[\begin{array}{c} S_{k,1}^{*}\cdot N_{k,n}\\ S_{k,2}^{*}\cdot N_{k,n}\\ \vdots \\ S_{k,M}^{*}\cdot N_{k,n} \end{array}\right]$$

And the STC-decoded accumulated echo is

(22) $$S_{D_{n}}^{l}\left(f_{r}\right)=\left[\begin{array}{c} H_{1,n}^{l}\cdot \sum_{k=1}^{K}{\left|S_{k,1}^{l}\right|}^{2}\\ H_{2,n}^{l}\cdot \sum_{k=1}^{K}{\left|S_{k,2}^{l}\right|}^{2}\\ \vdots \\ H_{M,n}^{l}\cdot \sum_{k=1}^{K}{\left|S_{k,M}^{l}\right|}^{2} \end{array}\right]+\left[\begin{array}{c} \sum_{k=1}^{K}S_{k,1}^{l}\cdot N_{k,n}^{l}\\ \sum_{k=1}^{K}S_{k,2}^{l}\cdot N_{k,n}^{l}\\ \vdots \\ \sum_{k=1}^{K}S_{k,M}^{l}\cdot N_{k,n}^{l} \end{array}\right]$$

After STC decoding, the echo level is *√K* times the echo level of a conventional MIMO-SAR system after pulse compression, while the noise level of the former is *K* times that of the latter. This means that, relative to a traditional MIMO-SAR system, STC can provide a maximum echo gain of 10 × *log*10(*K*) dB.

### OFDM Waveform Design

For the selection of specific transmitted waveforms, it is necessary to consider the characteristics and requirements of the aforementioned coding schemes, ensuring a certain degree of flexibility and scalability while minimizing negative impacts on imaging. This allows for better high-quality separation and processing of MIMO-SAR echoes. The STC scheme proposed in this paper is designed for coding matrices of arbitrary dimensions and involves transmitting different waveforms from multiple transmit units. However, in practical applications, carrier motion and trajectory variations may lead to decoding inaccuracies, resulting in autocorrelation and cross-correlation interference. Particularly, when multiple transmit units operate simultaneously, the similarity between the transmitted signals may cause significant interference:

(23) $$S_{D}^{\prime }=DR=S^{\prime }+S_{cross}^{\prime }=\left({\left|S_{1}\right|}^{2}+{\left|S_{2}\right|}^{2}\right)H_{1}-\left({\left|S_{2}\right|}^{2}+{\left|S_{1}\right|}^{2}\right)H_{2}+S_{1}S_{2}\Delta H_{1}+S_{1}S_{2}\Delta H_{2}$$

**Figure 4:**
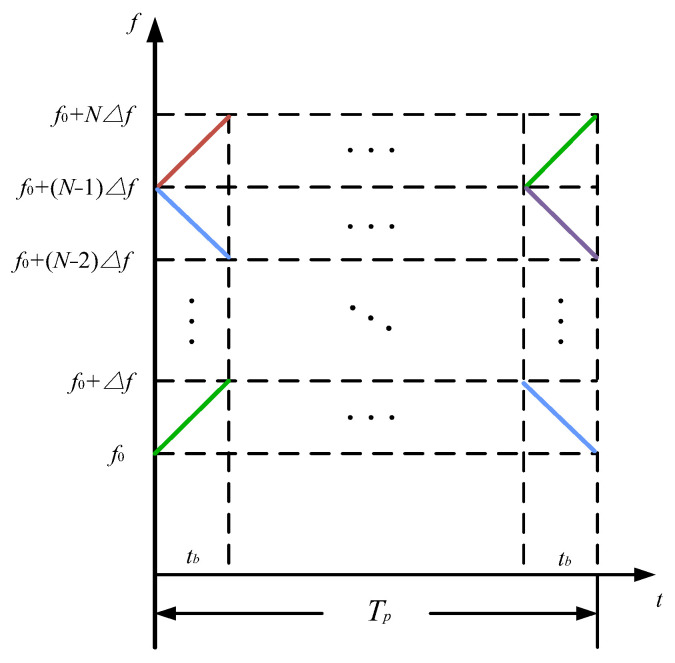
Time−frequency structure of OFDM−LFM waveform.

To address scenarios with more than two transmit units, as described in this paper, waveforms with higher flexibility and a greater number are required. In this context, OFDM waveforms, known for their high design flexibility and widely used in radar and communication systems, offer a suitable solution. By combining LFM with OFDM, and dividing the transmission time and bandwidth into non-overlapping segments, the OFDM-LFM waveform is created. This waveform provides greater design flexibility and orthogonality, making it more suitable for radar systems while effectively mitigating interference when multiple transmit units operate simultaneously. Typically, the more subcarriers the OFDM waveform is divided into, the lower the level of mutual correlation. The time–frequency diagram of this waveform is shown in Figure 4.

In this study, to ensure the orthogonality of the waveforms and reduce self- and mutual correlation interference, the modulation matrix of the time–frequency structure is optimized to improve the performance of the waveforms.

The time-domain structure of a single OFDM-LFM waveform is as follows:

(24) $$s(t)=\sum_{n=0}^{N-1}\sum_{m=0}^{M-1}a_{n}\cdot \exp\left\{j2\pi f_{n,m}t_{n}+j\pi k_{n}t_{n}^{2}\right\}$$

In this context, *f_n,m_* and *k_n,m_* represent the subcarrier frequency and the chirp rate of the sub-chirp corresponding to the element in the *n*-th row and *m*-th column of the matrix, respectively, for *u* (*t*) = 1, 0 < *t* < *T_b_*. To generate the required OFDM-LFM waveforms, this study employs a modulation matrix to control the time–frequency structure of the OFDM-chirp waveforms, The basic modulation matrix of the OFDM-LFM waveform can be represented as follows:

(25) $$\Phi (n,m)=u(t-mT_{b})\exp\left\{j2\pi f_{n,m}(t-mT_{b})\right\}\times \exp\left\{j\pi f_{rn,m}{\left(t-mT_{b}\right)}^{2}\right\}$$

The modulation matrix Φ is an *N × M* dimensional matrix, where each element is independently represented by −1, 0, or 1. Here, 1 indicates that the corresponding position in the matrix corresponds to a chirp waveform with a positive slope, −1 indicates a chirp waveform with a negative slope, and 0 indicates that the corresponding chirp waveform is not used in the signal. The modulated OFDM-LFM waveform can be expressed as the sum of all the elements in the matrix Φ.

In order to ensure the quadrature performance of the waveform, the waveform needs to maintain a low level of autocorrelation and cross-correlation at the same time so as to construct the optimization function:

(26) $$\min\left\{\sum_{\begin{array}{c} p\neq 0\\ p=1-P \end{array}}^{P-1}\sum_{n=1}^{N_{t}}{\left|R_{n,n}(p)\right|}^{2}+\lambda \sum_{m=1}^{N_{t}}\sum_{n=1}^{N_{t}}\sum_{p=1-P}^{P-1}{\left|R_{m,n}(p)\right|}^{2}\right\}$$

**Figure 5:**
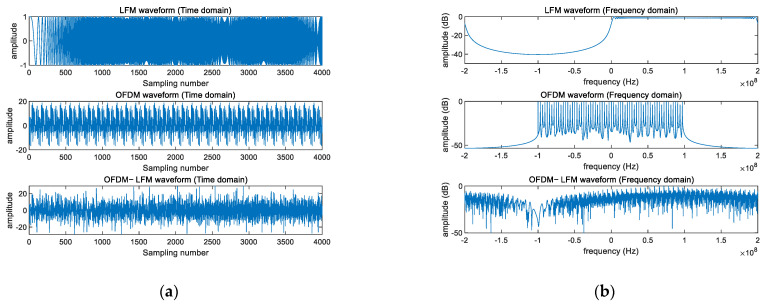
OFDM−LFM waveform generation. (**a**) Time dimension. (**b**) Frequency dimension.

The signal model, after being optimized for its autocorrelation and cross-correlation properties, yields waveforms suitable for the coding method discussed in this paper. The generation and representation of the OFDM-LFM signal waveform in both the time and frequency domains are shown in Figure 5.

**Figure 6:**
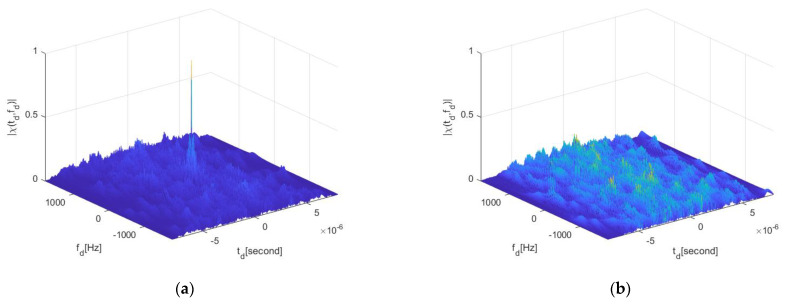
OFDM−LFM waveform ambiguity function. (**a**) Auto−ambiguity function for 16 subcarriers. (**b**) Cross−ambiguity function for 16 subcarriers.

For the OFDM-LFM waveform, the correlation performance improves as the number of subcarriers increases. The ambiguity function is shown in Figure 6, when the number of subcarriers is 16. It can be observed that the main lobe of the ambiguity function is clearer, presenting a peak shape, with lower side lobe levels and reduced peak values in the cross-ambiguity function. However, this comes at the cost of increased computational complexity.

**Figure 7:**
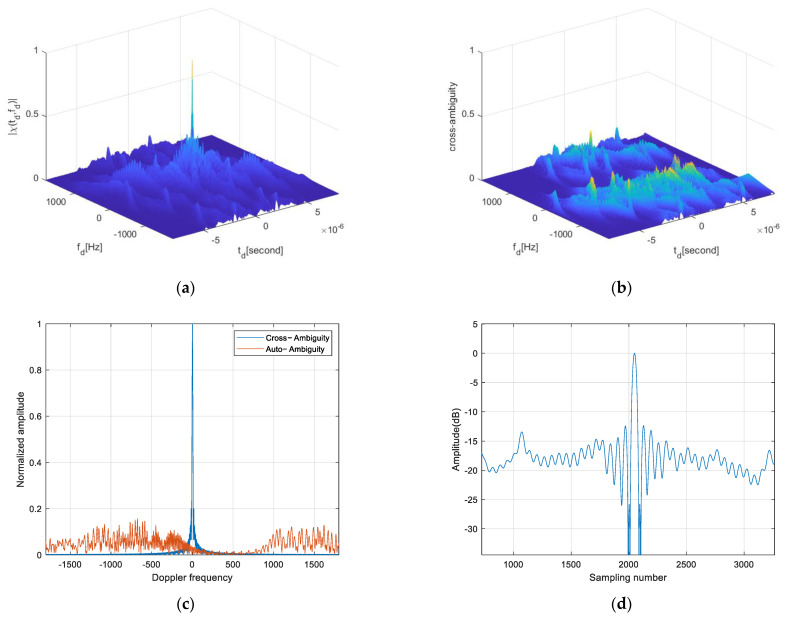
OFDM−LFM waveform ambiguity function. (**a**) Auto−ambiguity function for 8 subcarriers. (**b**) Cross−ambiguity function for 8 subcarriers. (**c**) Ambiguity function Doppler profile. (The blue line is autocorrelation and the orange line is cross-correlation) (**d**) Ambiguity function time profile.

To further analyze the performance of the waveform property, an OFDM waveform with eight subcarriers is used for ambiguity function and slicing calculations, as shown in Figure 7, which illustrates the main lobe width and side lobe levels.

**Figure 8:**
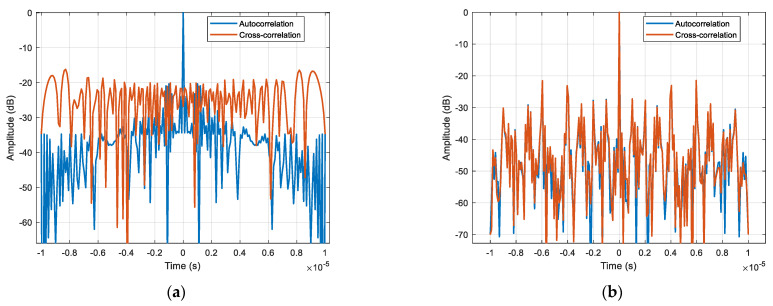
Waveform correlation function. (**a**) Up−down−chirp correlation function. (**b**) OFDM−LFM correlation function.

A comparison between the OFDM-LFM waveform and the up-down-chirp waveform is made. Up–down–chirp waveforms typically maintain orthogonality between two transmit units. However, when multiple transmit units are used simultaneously, transmitting identical signals at different times may cause autocorrelation and cross-correlation interference, which limits system performance. The side lobe levels and main lobe width of the two waveforms are shown in Figure 8, where the OFDM-LFM waveform exhibits better numerical characteristics. For cases where the number of waveforms exceeds 3, the cross-correlation ambiguity is evenly distributed across the time domain. This results in cross-correlation interference when imaging distributed targets, causing image blurring, thus making it difficult to meet the requirements of the STC scheme presented in this paper. In contrast, the OFDM wave-form exhibits lower side lobes in both its autocorrelation and cross-correlation as shown in Table 1, effectively reducing interference.

## 4. Experiment

This section validates the effectiveness of the proposed STC scheme through numerical experiments, which are divided into three parts. The first part evaluates the detection performance of the STC scheme to verify its capability in enhancing the echo SNR. The second part conducts SAR point target imaging experiments to analyze the focusing performance achieved by the proposed scheme using OFDM waveforms. The third part involves simulated imaging of distributed targets to demonstrate the superiority of the proposed STC scheme in imaging quality compared to traditional orthogonal waveform-based echo separation methods in MIMO-SAR systems.

### 4.1. STC Performance

**Figure 9:**
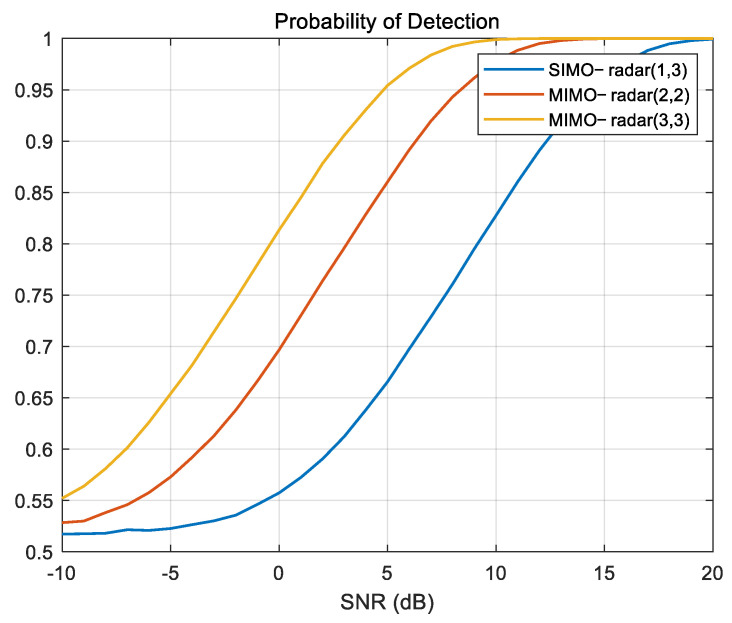
STC scheme detection performance.

This section investigates the enhancement of detection probability in MIMO-SAR systems through the application of the STC method. The radar parameters used in the experiment are shown in Table 2. The study employs the CFAR method with a detection threshold set to 10^−2^, comparing the detection probability of MIMO-SAR systems with 1, 2, and 3 transmitting units and 3 receiving units, respectively. The results, as presented in Figure 9, demonstrate a significant improvement in detection probability when the STC scheme is applied compared to the system without it. As the number of transmitting and receiving components increases, the performance improvement with STC becomes more noticeable, showing the effectiveness of the proposed method in enhancing detection capabilities.

**Figure 10:**
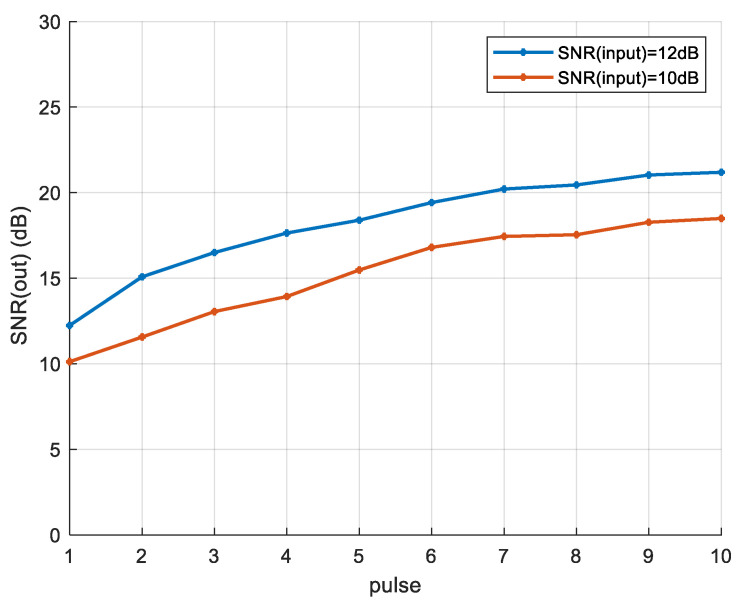
STC scheme SNR performance.

The performance of the STC scheme under different SNR conditions is shown in Figure 10. STC scheme SNR performance. Under input SNR conditions of 10 dB and 12 dB, the STC scheme demonstrates a certain level of SNR improvement. Moreover, as the size of the encoding matrix and the number of processing pulses increase, the improvement in SNR becomes more pronounced. Further, the characteristics and advantages/disadvantages of the MIMO-SAR STC schemes can be compared in detail in Table 3.

### 4.2. Point Target Simulation

The second part of this study evaluates the point target imaging performance of the proposed scheme. The radar parameters are detailed in Table 4.

**Figure 11:**
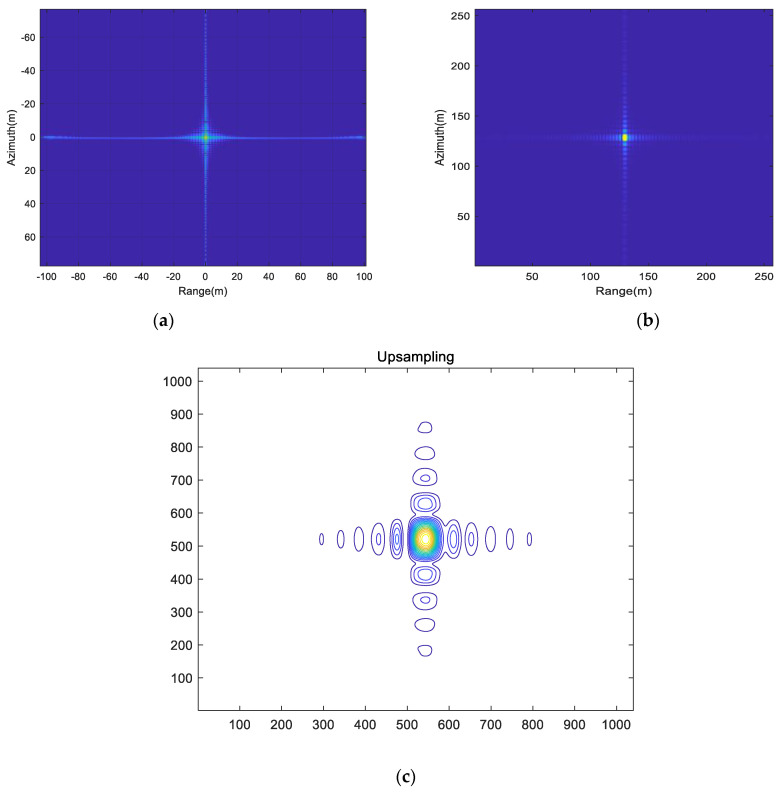

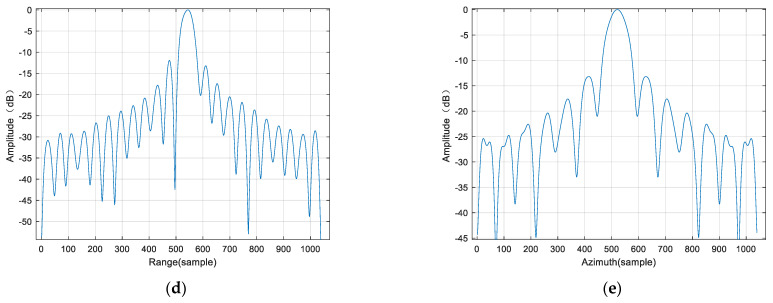
Point target imaging results. (**a**) Imaging results of the point target scene according to up−down−chirp. (**b**) Imaging results of the point-target scene according to OFDM−LFM. (**c**) Upsampling point imaging result. (**d**) Range profile. (**e**) Azimuth profile.

The MIMO-SAR transceiver unit used in the simulation is 3, and the simulation parameters are based on conventional airborne SAR. The focused imaging results for point targets are illustrated in Figure 11, accompanied by the range and azimuth cross-sections. It can be observed that, with the traditional scheme using up–down–chirp waveforms, the point target experiences scattered energy. In contrast, through the method proposed in this paper, the point targets can achieve imaging results that are free of aliasing interference. The imaging effect of point target is clear, its index can meet the requirements of waveform design, and it is basically consistent with the sidelobe level of conventional LFM signal, which can meet the imaging requirements.

### 4.3. Scene Simulation

**Figure 12:**
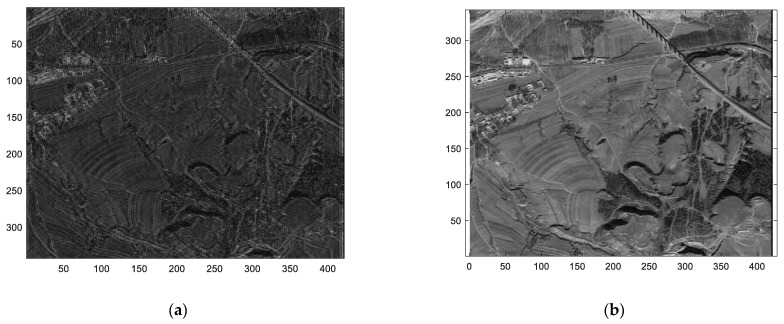
Distributed target imaging result. (**a**) Up–down–chirp STC scheme. (**b**) OFDM-LFM STC scheme.

**Figure 13:**
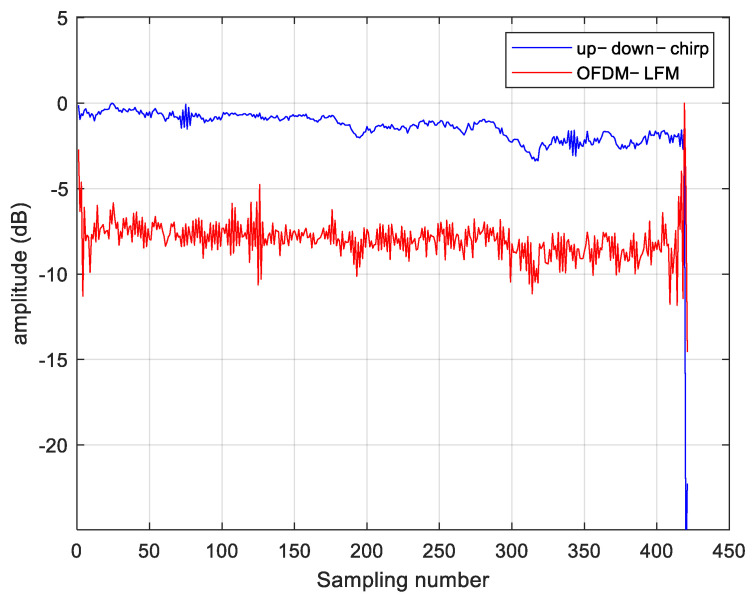
Azimuth averaged ambiguous energy.

To further validate the proposed STC-OFDM scheme, scenario-based target simulation experiments were conducted. A comparative analysis with up–down–chirp waveforms revealed that, while LFM signals can be processed in MIMO systems via matched filtering, they exhibit a certain level of cross-correlation interference throughout the entire duration. This interference accumulates as the imaging process progresses, ultimately leading to a significant degradation in image quality. In contrast, the method proposed in this paper consistently maintains a low level of cross-correlation interference during processing, effectively reducing the accumulation of interference and generating clear, high-quality images. The experimental results in Figure 12 and Figure 13 show the feasibility and effectiveness of the proposed STC encoding scheme and OFDM waveform in practical applications.

## 5. Conclusions

This study explores the integration of MIMO-SAR and communication system technologies, proposing a STC scheme based on complex orthogonal space–time block codes to address the echo separation problem in MIMO-SAR systems. The inherent similarities between radar and communication systems provide a promising avenue for the application of communication technologies in radar. However, due to the need for high-resolution imaging of ground objects in SAR systems, integrating communication technology into SAR presents significant challenges. Therefore, this study analyzes the MIMO-SAR signal model and discusses the key characteristics and advantages of applying STC technology in this context.

Building on the basic principles of traditional STC encoding, especially Alamouti encoding, a novel complex orthogonal space–time coding scheme is proposed. This scheme overcomes the limitations of traditional STC in terms of the number of transmit units and is capable of constructing space–time encoding matrices of arbitrary dimensions, thereby expanding the degrees of freedom of the waveform scheme. Additionally, by integrating OFDM-LFM waveforms, the proposed scheme effectively reduces correlation interference, thereby further enhancing the overall performance of the MIMO-SAR system. However, in practical applications, the proposed solution requires additional transmission pulses for encoding and decoding processing, which places higher demands on the operational accuracy of the SAR system.

Through the flexible application of communication technologies, STC not only addresses the echo separation problem in MIMO-SAR but also holds potential for solving various issues in radar-communication integration, including encoding and communication challenges. The use of more subcarriers and lower correlation interference in the OFDM waveform results in higher waveform complexity, which contributes to its potential for enhanced interference resilience.

Future research will focus on the limitations of STC technology in terms of the pulse count and transceiver frequency, as well as the impact of the encoding matrix size on MIMO-SAR imaging processing. Further exploration will be directed at optimizing encoding transmission efficiency, reducing interference caused by channel variations, and examining the Doppler performance impact of OFDM waveforms in the MIMO-SAR imaging process. By introducing more advanced encoding and optimization methods, the performance of the waveform scheme may be further improved.

## Figures and Tables

**Table 1:** Correlation function parameters.

Waveform	IRW (Sample)	PSLR (dB)	ISLR (dB)
Up–down–chirp	0.89	−12.87	−10.31
OFDM-LFM	0.9	−12.73	−11.86

**Table 2:** STC radar parameters.

Parameter	Value
Carrier frequency	9 GHz
Platform speed	600 m/s
Platform height	10 km
Pulse width	10 µs
Bandwidth	6 MHz
Sampling frequency	50 MHz
Number of transmitters	1, 2, 3
Number of receivers	2, 3
*P_fa_*	10^−2^

**Table 3:** Comparison of different MIMO-SAR schemes.

Scheme	Separation	Advantages	Drawbacks
Improved Alamouti in [18]	Range-Doppler domain	Avoids time-varying channel interference	Coding dimension is 2 SNR loss
Improved Alamouti in [19]	DBF inelevation range	Channel interference mutual cancelation	Coding dimension is 2 DBF interference
STC scheme in [17]	Slow-time domain	Decreases the sidelobe ratio	Coding dimension for even
STC scheme in [21]	Range-Doppler domain	Arbitrary coding dimension Avoids time-varying interference	PRF > N∙Ba
STC scheme proposed in this paper	Slow-time domain	Arbitrary coding dimension Reduces correlation interference	More processing pulses

**Table 4:** SAR imaging parameters.

Parameter	Value
Carrier frequency	10 GHz
Platform speed	150 m/s
Platform height	20 km
Pulse width	20 μs
Chirp Length	2.5 μs
Number of Carriers	8
Bandwidth	50 MHz
PRF	500 Hz
Antenna Length	1 m
Number of transmitters	3

## Data Availability

No new data were created or analyzed in this study. Data sharing is not applicable to this article.
